# Metallurgical and Hydrogen Effects on the Small Punch Tested Mechanical Properties of PH-13-8Mo Stainless Steel

**DOI:** 10.3390/ma11101966

**Published:** 2018-10-12

**Authors:** Yoav Snir, Shlomo Haroush, Albert Danon, Alex Landau, Yaniv Gelbstein, Dan Eliezer

**Affiliations:** 1 Department of Materials Engineering, Ben-Gurion University of the Negev, Beer-Sheva P.O.B 653, Israel; snirys@gmail.com (Y.S.); dan.eliezer@gmail.com (D.E.); 2 Department of Materials Science, Nuclear Research Center Negev, Beer-Sheva P.O.B. 9001, Israel; monih6655@gmail.com (S.H.); albdanon@yahoo.com (A.D.); alandau11@012.net.il (A.L.)

**Keywords:** PH-13-8-Mo, hydrogen embrittlement, small punch test

## Abstract

PH13-8Mo is a precipitation hardened martensitic stainless steel, known for its high strength but also for its high sensitivity to hydrogen embrittlement. Small punch test, SPT (also referred to as the ball punch test, BPT), is a relatively simple and new technique to assess the mechanical properties of samples under biaxial loading conditions. The current study utilizes the unique loading conditions of SPT to investigate the mechanical behavior and fracture prior to and after the hydrogen charging of PH13-8Mo steel. The mechanical characteristics were investigated at different metallurgical conditions: solution and quenched (SQ); fully-aged (550 °C for 4 h) and over-aged (600 °C for 4 h). Samples were cathodically hydrogen charged in a 1 M H_2_SO_4_ solution containing NaAsO_2_ (0.125 mg/L) at 50 mA/cm^2^ for different durations of 0.5 h, 2 h, and 19 h, and compared to the as-heat-treated condition. A fractographic examination was performed following the SPT measurements by scanning electron microscopy (SEM). Transmission electron microscopy (TEM) and x-ray diffraction (XRD) analyses were used as complementary characterization tools. It is shown that upon hydrogen charging, the SPT fracture mode changes from ductile to completely brittle with a transition of mixed mode cracking also affecting the SPT load-displacement curve.

## 1. Introduction

Precipitation hardened martensitic stainless steels (PHMSS) are an important sub-class of the Fe-Cr-Ni type alloys, which are widely utilized in the aerospace and petrochemical industries due to their optimized combination of high strength, toughness, and good corrosion resistance [1,2]. PH 17-4, PH 15-5, the Custom 4xx series and PH-13-8-Mo are commercial examples of these steels. Common to these alloys is the ability to heat treat them by, first, a solution treatment to gain a martensitic matrix followed by different aging treatments, similar to maraging steels. These treatments usually create different amounts of strengthening precipitates, intermetallic or metallic phases and some amounts of reverted austenite γ-FCC phase [1,2,3,4,5,6]. Another common characteristic of PHMSS is their extremely high sensitivity to hydrogen induced cracking at their peak-aged condition which was extensively shown [2,3,4,7,8,9,10,11,12,13,14,15,16]. Yet, like other steel alloys, the effects of hydrogen on the mechanical properties, e.g., yield strength, tensile strength, under different testing conditions and metallurgical conditions are still non-conclusive [15,16,17]. This is mainly due to the inherent complexity of the hydrogen effects themselves combined with the multi-parametric nature of studies which combine different testing methods, different hydrogen charging conditions, and different metallurgical conditions. All of these can have a dramatic effect on the results [7,17]. Therefore there is a continuous effort to further develop new, simple, yet highly sensitive methods to characterize these effects.

PH-13-8Mo is not an exception in this sense. The nominal composition of the alloy is Fe-13wt.%Cr-8wt.%Ni-1wt.%Al-2wt.%Mo [1]. In the as-solution-and-quenched (SQ) condition, a matrix of soft lath martensitic α-Fe (BCC, *a* = 0.2866 nm) is dominant [6,18]. The Al content enables the precipitation of coherent ordered β-NiAl (B2, *a* = 0.2887 nm) through age hardening treatments [18,19,20,21,22] thus giving it a BCC-BCC’ strengthening mechanism which is highly resistant to coarsening even at temperatures as high as 650 °C [6,18]. The aging treatments are usually performed at a temperature range of 500–620 °C for a duration of 4 h, termed H-xxxx treatments, yielding ultra-high mechanical properties (up to 1620 MPa), but also a certain amount of γ-FCC reverted austenite upon aging above 550 °C [1,23,24,25]. Reverted austenite in this alloy is mainly formed through a combination of diffusional processes at lath boundaries in the alloy yielding a local (nanometer sized) Ni enrichment stabilizing the austenite phase. Its presence contributes to a higher fracture toughness and it is also claimed to improve the resistance to hydrogen embrittlement [8,9,10,11,12,13,14,15,16]. 

**Figure 1:**
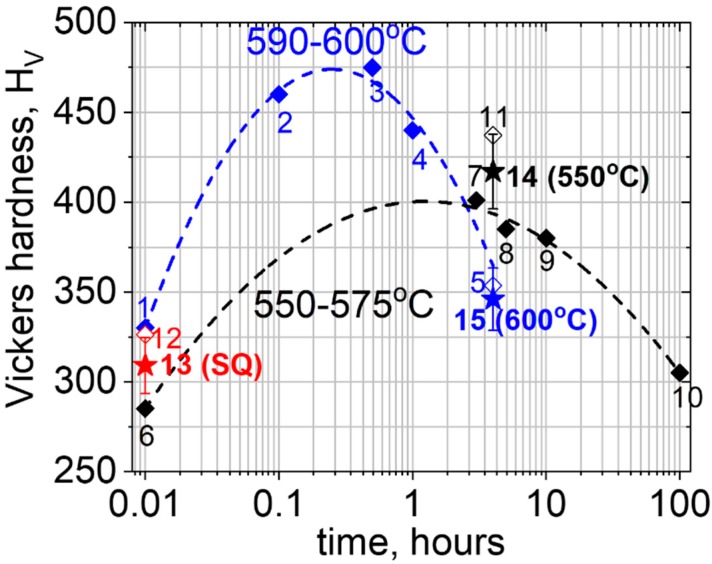
The Vickers hardness variation over time for PH-13-8Mo stainless steel as was previously reported for aging at 590–600 °C (blue points 1–4 [18]; 5: an average value from References [1,6,10,13,18,19,20,21,22] and curve) and 550–575 °C (black points 6–10 [24]; 11: an average value from References [1,6,10,13,18,19,20,21,22] and curve) as well as the variation following solution and quenching treatment (red point 12 [1,6]). The currently measured points under the investigated thermal treatment conditions are shown as the star points 11 (550 °C), 15 (600 °C), and 13 (following solution and quenching).

PH-13-8Mo was extensively studied in recent years in terms of its metallurgical and mechanical behavior, including the precipitation sequence as manifested by hardness tests, and the formation and stability (mechanical and thermal) of reverted austenite (γ-FCC) [23,24,25]. As can be seen in Figure 1, hardness values following aging durations of this steel have been published [1,6,10,13,18,19,20,21,22,24]. As can be seen in this figure, previously reported hardness variation values with the aging time at the two temperature ranges of 590–600 °C (blue points and curve) and 550–575 °C (black points and curve), had shown that the maximal aging conditions for the former were ~0.2–0.7 h, and ~1–6 h for the latter. A longer aging duration results in over-aging and decreased hardness values. Based on these observations, a 4 h aging duration was established as an accepted standard aging condition for both of these temperature ranges [1,6,10,13,18,19,20,21,22], while considering that this period of time is equivalent for maximal aging for a temperature range of 550–575 °C, while over-aging conditions will occur at temperatures of around 600 °C. Due to this standardization, most of the practically applied PH-13-8Mo stainless steel components are being aged at these temperatures for 4 h, prior to being subjected to practical environmental conditions, including hydrogen exposure.

## 2. The Small Punch Test (SPT)

The use of the small punch test (SPT) in recent years has gained increasing attention as a quasi-nondestructive high throughput technique to assess the mechanical properties of metals and alloys [26,27,28,29,30,31,32,33,34]. This method had been first established for radiation damage characterization in steels [26] and even standardized to some extent [27,28], especially to assess mechanical properties of small dimensional samples [29,30,31,32]. These properties include the yield strength, ultimate tensile strength, toughness and strain energy [32,33,34].

The SPT technique concept is based on locking a thin sheet-like specimen between two dies and pushing a spherical punch against it until failure. During the test, the load and the punch stroke are monitored simultaneously until the end test criterion (e.g., maximal or failure load or a certain stroke) is achieved. The ASTM standard E-643 and others [27] address specimen thicknesses between 200 and 2000 µm. Indeed, most of the reported studies were focused on alloys that have a thickness larger than 200 μm [32]. Moreover, in the last decade, most of the academic research was focused on specimens with a thickness of 500 μm, as the European Committee for Standardization Code of Practice specifies [28].

The main challenge in SPT is to extract the mechanical characteristics of the material, such as the yield stress and ultimate stress that are commonly the goal of standard tension tests. In this method, typical load-displacement curves contain four regions, described in References [28,30,35,36]: I = Elastic behavior region, II = Plastic behavior (strain strengthening) region, III = Plastic membrane stretching region, and IV = plastic instability region. According to Reference [28], the typical SPT curve is a smooth curve and should not contain any irregularities (discontinuities, “pop ins”, etc.).

In the vicinity of the maximal load, cracks are expected to develop in the specimen, followed by ductile “necking” propagation and fracture. The transition zone between regions I and II, which corresponds to the plastic yielding of the sample, is used to estimate the material yield stress, while the ultimate stress and fracture strain are estimated from zone IV.

The prediction of tensile strength and yield stress from the SPT test is based on the linear relationship existing between load and deflection (zone I) and the onset of deviating from it (zone II). In a recent study by Garcia et al. [32], different empirical correlation functions and data analysis approaches to the SPT curve were assessed. This was used to analyze SPT’s conducted on a variety of metallic alloys, including different types of steels having a wide range of mechanical properties, where all specimens had a thickness of 500 μm.

It was concluded by Garcia et al. [32] that the best estimation for the yield stress, *σ_ys_*, of a metallic specimen can be obtained by utilizing Equation (1) based on the *P*_*y_t*/10_ method shown in his work.

(1) σys=α1·Pyt2

where, the parameters in the equation are *P_y_*, the load in the SPT curve; *t*, the specimen thickness; *α*_1_, a unitless slope parameter. Garcia et al. [32] determined *α*_1_ to be equal to 0.346, providing a good approximation for all the tested materials.

Up to date, only a limited number of studies (e.g., Komazaki et al. [37]) have shown the effects of aging/precipitation hardening treatment in steels on the SPT behavior. Additionally, the SPT method has been only scarcely applied to assess hydrogen effects in steels [37,38,39,40,41]. Garcia et al. [40,41] and Komazaki et al. [37] have demonstrated the potential of SPT for hydrogen embrittlement related studies. Depending on the hydrogen charging conditions of SPT samples, it is expected that near-surface zones are much more affected than the bulk, dependent on the potential of SPT to more accurately analyze the mechanical behavior in these areas, compared to classic bulk measurement methods, averaging the mechanical properties over the entire bulk. Moreover, an SPT’s relative simplicity, and the fact that charging conditions can be easily controlled (small thin samples), sample dimension and loading conditions possibly create better sensitivity to the hydrogen-rich layer in electrochemically charged samples.

## 3. Hydrogen Effects on PH13-8Mo

As was mentioned, hydrogen embrittlement in high strength steels is a very complicated phenomenon and so is the case for PH13-8Mo. It is important to note that while, in general, and also for PH13-8Mo, most studies of hydrogen effects on the mechanical properties were focused on uniaxial or triaxial loading conditions, i.e., the standard dog-bone shape sample or shaped notched specimens, hydrogen embrittlement studies under biaxial loading are scarce. Yet, for example, in metal forming processes [42] or in pipes and cylindrically shaped tanks, biaxial loading is common and might control the deformation behavior. In this sense, the unique loading path applied during SPT enables an investigation of the associated biaxial straining condition in a very simple and controlled manner.

As mentioned above, specimens of uniaxial [8,9,10,15,16] or notch geometry [11,12] were used in recent years to study PH13-8Mo. These studies showed that hydrogen slightly increased the yield strength but mainly decreased the elongation to the fracture and fracture toughness at different metallurgical conditions. These studies were each performed under single charging conditions. Specifically, it was claimed that a higher relative amount of reverted austenite, as a strong trapping site and plastic deformation promoter, could lead to a higher resistance to hydrogen embrittlement (e.g., References [8,9,10,11,12]). Even though this claim is understood in terms of the larger amount of the ductile γ-FCC phase, it is still non-conclusive with respect to the accumulation and distribution of hydrogen in the sample and its role in the fracture mechanism.

The aging conditions for the current study were not only chosen for identifying the mechanical variations between them, but also due to the expected drastic difference in hydrogen diffusivity and solubility following aging at 550 °C/4 h and 600 °C/4 h respectively. According to References [11,12] hydrogen diffusivity is reduced by an order of magnitude (from ~10^−12^ to ~10^−13^ [m^2^/s]) and hydrogen solubility is increased by an order of magnitude (from ~500 to ~3000 mol(H)/m^3^) by changing the aging conditions from 550 °C/4 h to 600 °C/4 h respectively.

In the current research, the unique SPT loading conditions were employed for a better understanding of the fracture modes and the mechanical properties of PH13-8Mo before and after hydrogen charging. For this purpose, the effect of the metallurgical microstructure on the mechanical properties are first examined via SPT, and secondly, the effect of charging duration on the mechanical behavior of PH13-8Mo steel is determined with an emphasis on the reverted austenite role on the load-displacement behavior and fracture under biaxial SPT loading.

## 4. Experimental

A commercial grade PH-13-8Mo rod was cut into 8 mm × 8 mm × ~0.7 mm thick disc samples with an electron-discharge machine (EDM). Each disc was first given a solution treatment, based on Reference [43] at 940 ± 5 °C for 2 h under an inert-gas-environment and a subsequent oil quenching followed by a soaking in iced water for at least 1 h to ensure full martensitic microstructure. Following the solution and quenching (SQ) treatment, the samples were aged at 550 °C and 600 °C for 4 h followed by a water quench, indicating the solution treatment for the fully-aged and over-aged conditions. These conditions also represent different relative amounts of the β-NiAl and γ-FCC phases (see Table 1) and were chosen for correlating the mechanical properties and hydrogen diffusivities with the microstructural characteristics as was mentioned earlier. Hydrogen charging was performed for different durations of 0.5, 2, and 19 h, following a 600 °C aging. Electrochemical cathodic (EC) hydrogen charging was applied in a solution of H_2_SO_4_ + NaAsO_2_ (1 M + 0.125 g/L respectively as a poisoning agent) at 50 mA/cm^2^ for the different durations. Each SPT sample was spot welded on the corner to a thin Pt wire which was connected to the DC-charge unit. A Pt-stripe served as an anode and was also connected to the DC-charge unit. Prior to hydrogen charging, each sample was carefully mechanically polished down to 1200 grit. Subsequently, in order to ensure a better surface finish, a final polish with 5 μm and 1 μm standard diamond pastes, respectively, was performed. The measured final thickness for each sample was in the range of 0.500 ± 0.015 mm.

Small punch tests, SPT, were performed on the investigated (8 × 8 × 0.5 mm^3^) samples using the same apparatus as described in References [33,34], with a 2.4 mm diameter hard steel ball which was lubricated prior to testing in order to avoid any friction effects during the test. All tests were performed at a stroke control of 0.1 mm/min and a 2.5 kN clamping load. This rather slow rate was chosen in order to better capture any possible effects of hydrogen on the SPT experiment in the early stage (zone I and II in the typical SPT curve). Each test was stopped as 80% of the maximal load (*F*_max_) was reached, for preserving the general features of the deformation and possible cracking. In order to ensure minimal hydrogen egress from the SPT sample, the hydrogen-charged samples were analyzed in the time range of not more than 15 min after terminating the EC charging duration.

Vickers micro-hardness testing was conducted on the polished samples under 200 g load for 10 s. Prior to testing, a standard block was used to verify the calibration of the testing machine. A minimum of 5 indents were taken from each sample.

X-ray diffraction (XRD) analyses following the solution treatment, full-aging and over-aging treatments were performed in the 20–100° angle range using a Cu-Kα source. The XRD patterns were fully analyzed by the WinPlot-2011 software.

Transmission electron microscopy (200 KV TEM, JEOL) samples were prepared from 3 mm × 0.1 mm discs by conventional mechanical grinding and polishing to a 1 µm final polish and ~25 µm thickness, followed by precision ion polishing (PIPS). Samples for scanning electron microscopy (SEM, JEOL JSM 5600, JEOL, Welwyn Garden, UK) fractographic examination were taken from the SPT to compare the general modes of failure, and the microcracking features between the different metallurgical and charging conditions.

## 5. Results and Discussion

**Figure 2:**
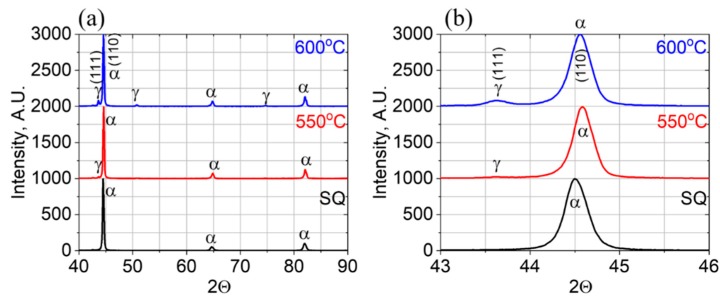
The XRD reflections of the investigated PH13-8 samples at different thermal treatments of solution and quenching (SQ) and aging at 550 °C and 600 °C on the wide 2Θ angle range of 40–90° (**a**) and in the vicinity of the the γ-(111) reflection at 2Θ ~ 43.7° (**b**).

XRD patterns following the different investigated thermal treatment conditions of solution and quenching (SQ) and aging at 550 °C and 600 °C (Table 1), are shown in Figure 2. As expected, the SQ state revealed only the α-Fe BCC martensite phase, whereas the aging treatments also revealed a growing amount of γ-FCC austenite, as can be seen for example in Figure 2b, by the increased relative intensity of the γ-(111) reflections at 2Θ ~ 43.7°, by increasing the aging temperature from 550° (~2%) to 600 °C (~10%).

**Figure 3:**
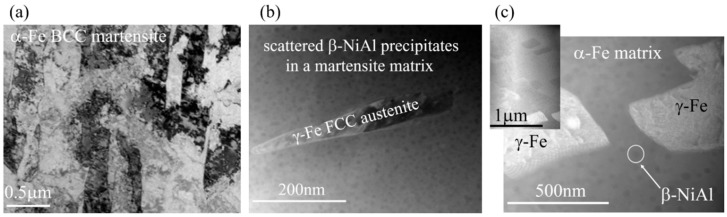
The TEM images following solution and quenching, SQ (**a**), and aging treatments at 550 °C (**b**) and 600 °C (**c**). Following SQ, only a BCC α-Fe martensite matrix is observed, while upon aging, the formation of a secondary γ-Fe FCC austenite phase surrounded by round β-NiAl precipitates can be clearly seen. HAADF analysis across the γ-Fe FCC austenite phase indicated an increase of Ni and a decrease of Al concentrations inside the austenite phase, as expected.

**Figure 4:**
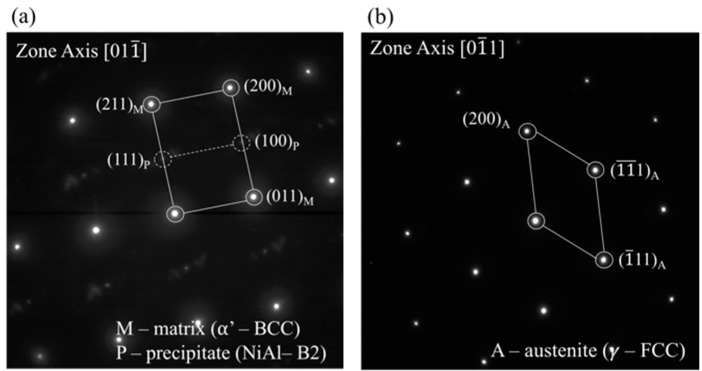
The selected area electron diffraction (SAED) patterns taken from the three phases shown in Figure 3. (**a**) Diffraction pattern from both the BCC α-Fe martensite matrix (M) and B2 β-NiAl precipitates (P), showing an orientation relation of [0$1\bar{1}$]_M_||[0$1\bar{1}$]_P_ and (200)_M_||(100)_P_ with a corresponding *a*_lattice_ = 2.898 Å. (**b**) Diffraction pattern taken from the FCC γ-Fe reverted austenite phase (A) with a corresponding *a*_lattice_ = 3.59 Å.

TEM images following the SQ, and aging at 550 °C and 600 °C are shown with representatively selected area electron diffractions (SAED) in Figure 3a–c and Figure 4a,b, respectively.

Figure 3a reveals a typical lath-martensite microstructure following SQ. No β-NiAl precipitates were found. Figure 3b shows the typical microstructure observed following the 550 °C aging treatment. Embedded β-NiAl precipitates in the martensite phase can be observed as was verified by the corresponding SAED patterns, Figure 4a. These small (less than 50 nm in diameter) precipitates were found to be below the detection point of the XRD (Figure 2). Yet, the shift of the α-Fe BCC martensite phase reflections toward higher 2Θ angles upon 550 °C aging, compared to the SQ condition, as can be seen for the α-Fe (110) reflection at 2Θ ~ 44.5° in Figure 2b, indicates a reduction of the martensite lattice parameter. Such a reduction of the lattice parameter of the martensite phase can be explained by the removal of the Ni and Al atoms upon the formation of these β-NiAl precipitates. Reverted austenite γ-FCC embryos, with an elongated (~400 nm in length and 10–40 nm in width) morphology, shown in Figure 3b, were scarcely found. It can be seen in the figure that the γ-FCC embryos are completely depleted of β-NiAl precipitates. The FCC structure was verified by selected area electron diffraction (SAED), Figure 4b, and the nominal higher Ni and lower Al concentrations of the austenitic γ phase, with respect to the surrounding matrix, were verified by a high-angle annular dark-field scanning (HAADF) mode analysis.

A typical microstructure following an aging treatment of 600 °C is shown in Figure 3c. This microstructure is comprised of an α-Fe martensite matrix embedded with round β-NiAl precipitates and Ni-rich reverted austenite (γ-FCC) islands. These reverted austenite islands exhibit a blocky-type morphology and are larger (~450 × 450 nm^2^) than the embryos obtained following aging at 550 °C (Figure 3b), in agreement with the XRD reflections analysis (Figure 2) and previous studies [18,19,23,24,25].

**Figure 5:**
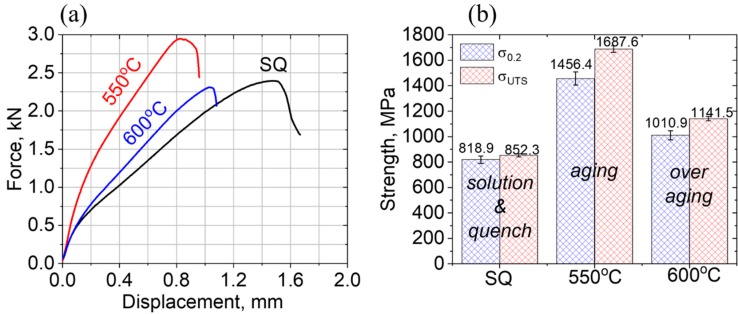
(**a**) The SPT load-displacement curves of the investigated PH13-8Mo samples, following solution and quenching (SQ) and aging treatments at 550 and 600 °C. (**b**) Yield (blue), σ_0.2_, and ultimate tensile (red), σ_UTS_, stresses, as were analyzed according to the Garcia et al. [23] method, from the SPT measurement values of (**a**). The error bars shown in (**b**) are based on a standard deviation of 5 measured samples for each treatment, giving 818.9 ± 28.6, 1456.4 ± 50.9, 1010.9 ± 35.4 MPa and 852.3 ± 12.8, 1687.6 ± 25.3, 1141.5 ± 17.1 MPa, corresponding to ~±3.5% and ~±1.5%, and the measurement errors for σ_0.2_ and σ_UTS_, respectively.

The measured small-punch-test, SPT, load-displacement curves, for the investigated uncharged PH13-8Mo, following SQ and aging treatments at 550 and 600 °C, are shown in Figure 5a,b.

These measured curves, shown in Figure 5a were analyzed by the *P*_t/10_ calculation method, previously reported by Garcia et al. [32], for obtaining the yield, σ_ys_, and ultimate tensile, σ_UTS_, strength values, shown in Figure 5b. Increasing trends of both σ_0.2_ and σ_UTS_ upon the aging of SQ PH13-8Mo steel at 550 °C (full-aging conditions) and the decreasing values at a higher temperature of 600 °C (over-aging), were obtained in agreement with previous standard uniaxial tensile mechanical testing observations [21,22]. A similar trend was observed following microhardness characterization, giving 309 ± 12 H_V_ (point 13 in Figure 1) following solution and quenching and 417 ± 16 (point 14 in Figure 1) and 346 ± 13 HV (point 15 in Figure 1), following 4 h aging treatments at 550 and 600 °C, respectively, in agreement with the previously reported values shown in Figure 1.

The obtained mechanical properties following the different aging conditions can be correlated to the microstructure evolution shown in Figure 3. Following SQ, the soft martensitic α-Fe BCC matrix yielded the highest elongation and lowest σ_0.2_ and σ_UTS_ values. Following full-aging at 550 °C, the evolution of the coherent β-NiAl precipitates and a small amount of γ-FCC reverted austenite, contributed to the strengthening of the steel, via precipitation hardening, while increasing both σ_0.2_ and σ_UTS_ and decreasing the displacement. Upon over-aging at 600 °C, an increase of the relative amount and the effective size morphology of the γ-FCC reverted austenite, on account of the β-NiAl precipitates was observed. It was reported before [23,24,25] that the evolution of the reverted austenite phase is accompanied by some dissolution of the β-NiAl precipitates, thus forming a local Ni-rich environment that promotes the formation of reverted austenite. Therefore, the effect of the reduced content level by dissolution and coarsening of β-NiAl precipitates reduces both σ_0.2_ and σ_UTS_. The increased displacement at this stage (600 °C), can be associated with the ductile nature of γ-FCC, which was reported before as a promoting agent for increasing the fracture toughness of PH13-8Mo [23,24,25].

**Figure 6:**
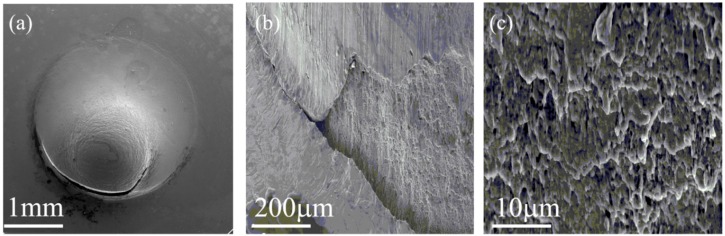
The SEM fractography micrographs at different magnifications following 600 °C over-aging. (**a**)—general view, (**b**,**c**)—higher magnifications.

These trends and the correlation with hardness measurements clearly demonstrate that the sensitivity of the SPT method, which was never applied before for PH13-8Mo, is adequate for distinguishing between the different aging conditions of the stainless steel. SEM fractography micrographs following SPT of a 600 °C over-aged sample, highlighting the ductile nature of the fracture, are shown in Figure 6.

As can be seen in Figure 6a, without any hydrogen charging, the failure begins as a semi-circumferential axial crack at the bottom of the ball punched bulb-shaped indentation as is typically observed in SPT experiments, e.g., References [28,31,32,33,34]. Higher magnifications (Figure 6b,c), highlight the ductile nature of the fracture, presenting an elongated dimple surface appearance.

**Figure 7:**
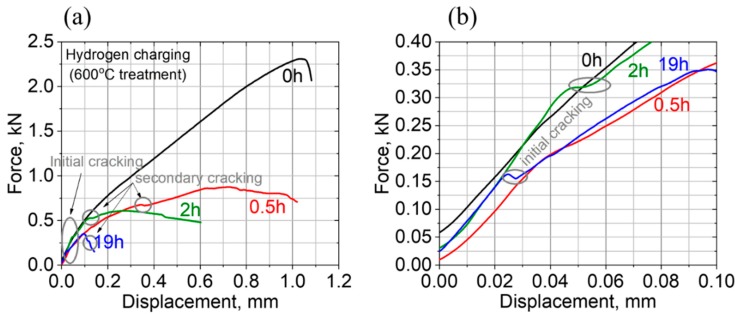
The SPT load–displacement curves for the investigated PH13-8Mo samples, following 600 °C aging treatment, prior and after electrochemical hydrogen charging for 0.5, 2, and 19 h in the entire examined displacement range (**a**) and at the 1st 0.1 mm displacement, where initial cracking was noticed following 2 and 19 h of hydrogen charging (**b**). Circle marks indicate cracking steps during SPT loading.

The effect of electrochemical hydrogen charging, for 0.5, 2, and 19 h. on the SPT curves following 600 °C over-aging, is shown in Figure 7a.

It can be clearly seen that both the maximal load and displacement values are monotonically decreased with increasing the hydrogen charging duration.

**Figure 8:**
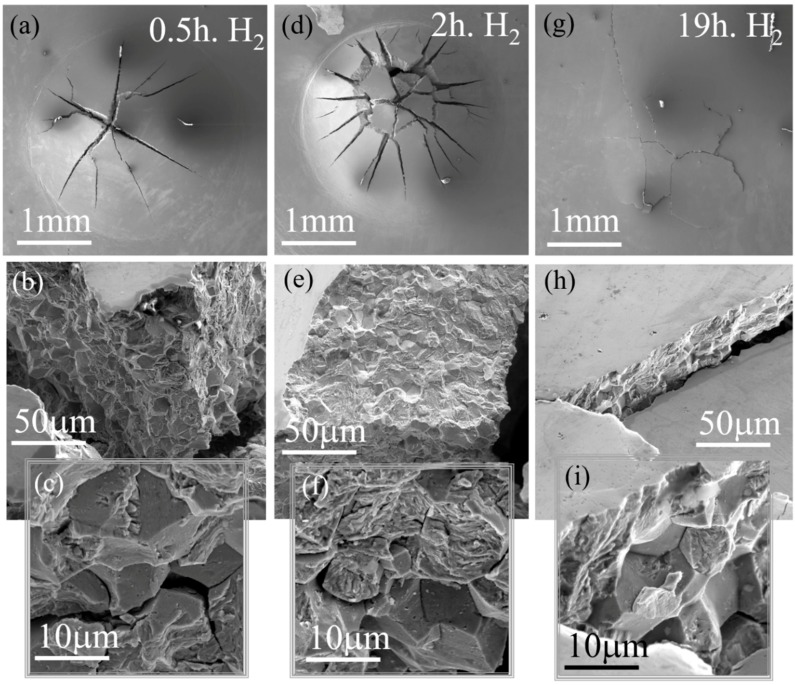
The SEM fractography micrographs following hydrogen electrochemical charging of 600 °C treated PH13-8Mo for 0.5 h (**a**–**c**), 2 h (**d**–**f**), and 19 h (**g**–**i**).

While the SPT curve of uncharged, 600 °C aged PH13-8Mo, shows maximal load and displacement values of ~2.4 kN and ~1 mm, respectively, these values decrease down to ~0.3 kN and 0.1 mm, respectively, following hydrogen charging for 19 h and the associated material’s embrittlement. Another interesting feature of these curve are the “pop-in” events (as indicated by arrows in Figure 7a) near the beginning of the stretching zone of the tested sample and during the last stretching zone. This serrated area indicates a ductile/brittle mixed-mode of deformation and is later validated by the SEM fractography images. While prior hydrogen to charging, Figure 5a, the SPT curves are smooth, the pop-in events, observed following hydrogen charging, can be correlated with the cracking of the tested specimens, upon hydrogen embrittlement. This “pop-in” phenomenon was also observed by Bruchhausen et al. [43,44,45,46] and interpreted in their case, as a crack initiation behavior during testing due to Ductile to Brittle Transition Temperature (DBTT) behavior. Therefore, in the current study, we can also interpret the pop-in appearance in Figure 7a,b as a crack initiation event related to the hydrogen effect, especially since their appearance is accompanied with the brittle fracture morphologies observed in Figure 8 and the fact that the uncharged SPT curves of the current samples were smooth. Additionally, it is important to note that all first “pop-in” events in the charged specimens appear at a smaller load compared to the uncharged specimens as can be observed in Figure 7b and this corresponds with increasing the hydrogen charging duration, as can be seen. The embrittlement evidence can be also seen by the SEM fractography micrographs following hydrogen charging for 0.5, 2, and 19 h, shown in Figure 8a–c, Figure 8d–f, and Figure 8g–i, respectively.

It can be clearly seen that in contrast to the typical bulb-shaped SPT indentation for ductile alloys, containing a semi-circumferential axial crack located at the lower part, as was observed for the uncharged 600 °C aged PH13-8Mo, Figure 6a, hydrogen charging, as depicted in Figure 8a,d, resulted in cracks initiated at the top of the SPT indentation.

Previous studies [33,34] have shown that during the SPT of ductile materials, cracks are initiated at the lower 1/3 of the height of the SPT indentation. This location suffers the highest stresses and thinning enabling crack formation. This failure mode was attributed to the unique biaxial stress distribution of the SPT [33]. Cracks initiation at the top of the SPT indentation, as is currently observed, following hydrogen charging, might hint at the involvement of a different stress distribution and additional strains field, interfering the plastic flow while creating the SPT indentation. Moreover, while the uncharged samples failed at the circumference of the developed indentation with a single circumferential crack, all the hydrogen-charged samples failed initially at the vertex of the cap with multiple radial cracks, indicating a brittle cracking nature.

Furthermore, after 19 h of charging, the typical SPT bulb-shaped deformation indentation observed in Figure 6a prior to charging is completely absent (Figure 8g), hinting again at the brittle characteristics of a premature fracture with a negligible plastic deformation. This behavior is in agreement with the respective SPT curve (Figure 7a), showing a very low energy to fracture (the small area under the curve).

Furthermore, at shorter hydrogen charging durations (e.g., 0.5 and 2 h), the higher maximal load and displacement values of ~0.8 kN, 0.7 mm and ~0.55 kN, 0.25 mm, respectively, (Figure 7a), were accompanied by a mixed brittle-ductile deformation nature. On one hand a bulb-shaped indentation, indicating a certain amount of plastic deformation, is noticed (Figure 8a,d), but on the other hand, it contains sharp radial cracks, initiating from the top of the indentation, which as was discussed above are generally indicative for a mechanically brittle fracture. This evidence is supported also by a higher magnification fractographic examination of the cracks. While prior to hydrogen charging (Figure 6b,c), fractographic examination revealed a dimple morphology, indicating a ductile behavior, a combined brittle intergranular fracture patterns of grain-boundary facets appeared upon intermediate charging (Figure 8b,c,e,f), and eventually transformed into a highly brittle-type morphology upon 19 h of charging (Figure 8h,i). The brittle fracture morphology contains some triple-junction intergranular cracks which are also observed (Figure 8c,f,i).

It should be noted that the effect of hydrogen on the tensile mechanical properties of PH13-8Mo was recently studied in References [15,16]. It was observed that hydrogen had a strong influence on the elongation to fracture and on the ultimate tensile stress, but the “pop-in” phenomenon was not observed. On the other hand, in the current study, the high sensitivity of the SPT technique enables us to identify both the influence of hydrogen on the mechanical properties and to show the initiation of cracking through the “pop-in” phenomenon. This is also supported by the fracture appearance seen in the SEM Fractography: Figure 6 and Figure 8.

The agreement between the fractography images and the SPT force-displacement curves, upon the hydrogen charging of over-aged PH13-8Mo for different durations, highlights the high potential of this mechanical characterization approach for analyzing near-surface hydrogen effects on the mechanical properties. The observed ductile to brittle transition upon the hydrogen charging of fully-aged PH13-8Mo, where the so-called hydrogen embrittlement resistant reverted the austenite phase, is highly developed and specifies the exact conditions in which this steel is capable of preserving the required mechanical properties under an externally applied hydrogen atmosphere.

## 6. Conclusions

The current research studies the effects of various metallurgical conditions of the solution and quenching and subsequent aging treatments, prior and following hydrogen charging, of PH13-8Mo maraging stainless steel on the mechanical properties evaluated by the small punch testing (SPT) method. From the SPT load-displacement curves, it was evident that maximal strengthening (and correspondingly hardening), indicating a full-aging condition, was observed following 550 °C aging for 4 h, while over-aging conditions with reduced strength values were obtained following 600 °C aging for the same period of time. The agreement of this trend with the phases and microstructure evolution, as was characterized by XRD and TEM, highlights the high sensitivity of the SPT method for monitoring the mechanical properties of the PH13-8Mo steel following various metallurgical conditions.

Cathodic hydrogen charging, for 0.5, 2, and 19 h, was used to introduce high fugacity hydrogen following over-aging at 600 °C for 4 h, where the relative amount and size of the γ-FCC reverted the austenite phase, reaching ~10% and ~450 × 450 nm^2^, respectively, were significant. In contrast to previous reports where it was implied that the reverted austenite is a strong hydrogen trapping site, by increasing the resistance to the hydrogen embrittlement of PH13-8Mo, the SPT following hydrogen charging indicated a transition from ductile to brittle behavior by increasing the charging duration. This finding, which is significant for practical applications involving PH13-8Mo in a hydrogen environment, could be observed very clearly due to the unique bi-axial loading characteristics of the SPT method. The unique characteristics of “pop-in” events during the SPT of hydrogen-charged samples enable a mechanical analysis of near-surface zones, which are much more affected than the bulk upon high-fugacity hydrogen charging, without averaging the mechanical properties over the entire bulk, as compared to standard mechanical characterization methods.

## Figures and Tables

**Table 1:** The PH13-8Mo samples’ designation and corresponding heat treatments expected from References [18,19,23,24,25].

Condition	Treatment	Microstructure/Phases
Solution and quenching (SQ)	940 °C/2 h oil quenching + 1 h soaking ice water.	α-Fe (Martensite)
Full-aged	SQ + 550 °C/4 h.	Martensite + β-NiAl + fine typically elongated (<400 × 10 nm^2^) γ-FCC (Austenite)
Over-aged	SQ + 600 °C/4 h.	Martensite + β-NiAl + coarser blocky type (~450 × 450 nm^2^) Austenite
